# Justifying the prediction of major soil nutrients levels (N, P, and K) in cabbage cultivation

**DOI:** 10.1016/j.mex.2024.102793

**Published:** 2024-06-04

**Authors:** Thilina Abekoon, Hirushan Sajindra, B.L.S.K. Buthpitiya, Namal Rathnayake, D.P.P. Meddage, Upaka Rathnayake

**Affiliations:** a Water Resources Management and Soft Computing Research Laboratory, Millennium City, Athurugiriya, 10150, Sri Lanka; b Department of Chemistry, Faculty of Science, University of Kelaniya, 11600, Sri Lanka; c Graduate School of Engineering, The University of Tokyo, Tokyo, 113-8654, Japan; d School of Engineering and Information Technology, University of New South Wales, Northcott Drive, Canberra, ACT 2612, Australia; e Department of Civil Engineering and Construction, Faculty of Engineering and Design, Atlantic Technological University, Sligo, F91 YW50, Ireland

**Keywords:** Cabbage cultivation, Deep neural network, Soil nutrient levels, TanSig function, Deep Neural Network (DNN)

## Abstract

In a recent paper by Sajindra et al. [1], the soil nutrient levels, specifically nitrogen, phosphorus, and potassium, in organic cabbage cultivation were predicted using a deep learning model. This model was designed with a total of four hidden layers, excluding the input and output layers, with each hidden layer meticulously crafted to contain ten nodes. The selection of the tangent sigmoid transfer function as the optimal activation function for the dataset was based on considerations such as the coefficient of correlation, mean squared error, and the accuracy of the predicted results. Throughout this study, the objective is to justify the tangent sigmoid transfer function and provide mathematical justification for the obtained results.

• This paper presents the comprehensive methodology for the development of deep neural network for predict the soil nutrient levels.
• Tangent Sigmoid transfer function usage is justified in predictions.
• Methodology can be adapted to any similar real-world scenarios.

## Graphical abstract

The Graphical Abstract for Justifying the Results of the Source Paper Published by the Authors [1].

**Figure:**
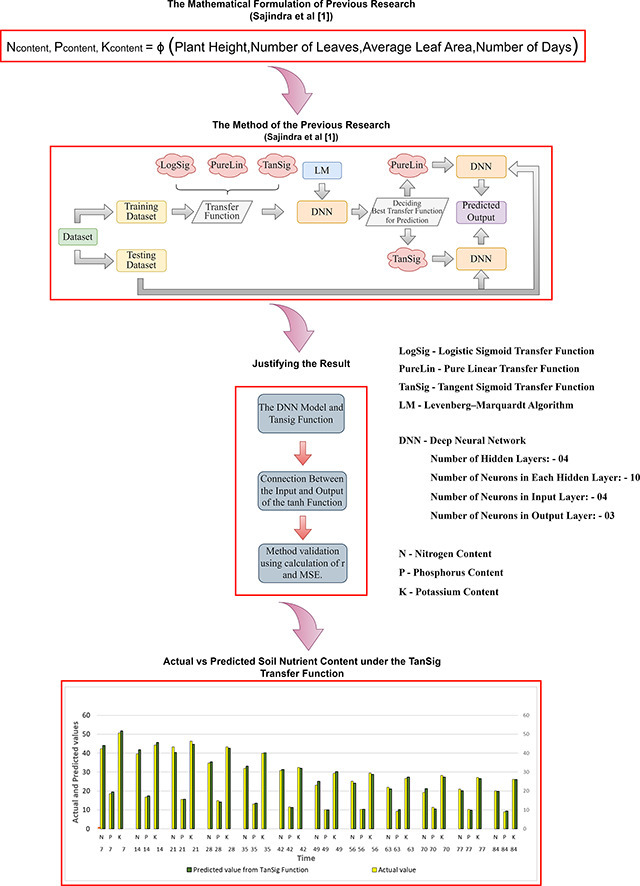

Specifications table

**Table:** 

Subject area:	Engineering
More specific subject area:	Machine Learning
Name of your method:	Deep Neural Network (DNN)
Name and reference of original method:	Sajindra, H., Abekoon, T., Jayakody, J. A. D. C. A., & Rathnayake, U. (2024). A novel deep learning model to predict the soil nutrient levels (N, P, and K) in cabbage cultivation. Smart Agricultural Technology, 7, 100,395. doi:10.1016/j.atech.2023.100395
Resource availability:	The data can be requested by corresponding author only for research purposes.

## Background

Sajindra et al. [1] have investigated the impact on the major nutrient contents of soil, specifically nitrogen (N), phosphorus (P), and potassium (K), in conjunction with the influence on the growth characteristics of cabbage plants, including plant height, number of leaves, and average leaf area. To identify the complex relationship between plant characteristics and soil nutrient content, a deep learning model was employed. Upon discerning the intricate relationships within the dataset, the Deep Neural Network (DNN) was used to predict soil major nutrient content with heightened accuracy [1]. The DNN model, characterized by its architecture consisting of four hidden layers each with ten nodes, underwent training using the Levenberg-Marquardt optimization algorithm. The training process resulted in the attainment of optimal performance metrics, specifically manifested in the lowest Mean Squared Error (MSE) values and elevated correlation coefficients (r) values. Notably, the application of the Tan*Sig* (Hyperbolic Tangent Sigmoid) transfer function during the training procedure contributed to achieving these favorable outcomes [2]. The utilization of *tansig*, known for its ability to model complex relationships, enhanced the DNN's capacity to capture intricate patterns in the data, thereby facilitating the attainment of superior performance metrics, which are essential indicators of the model's effectiveness in learning and generalization [3,4].

## Method details

### Dataset

The data for the model were acquired from three model agricultural farms of green coronet cabbage in Marassana, Nuwara Eliya in Central province, and Welimada in Uva province of Sri Lanka. Measurements were obtained systematically, commencing from the installation of germinated cabbage seeds and spanning 85 days, at 7-day intervals. Simultaneous measurements encompassed critical parameters including soil nutrient concentrations such as N, P, and K, (major soil nutrients) along with key plant growth indicators comprising Plant Height, Number of Leaves, and Average Leaf Area (refer to Table 1 for sample data). This comprehensive dataset, collected over a specific timeframe and at regular intervals, provides a robust foundation for a detailed analysis of the growth dynamics and nutrient interactions within the green coronet cabbage cultivation across different locations in the central hills of Sri Lanka [1].

**Table 1:** Sample data collected from organic cabbage cultivation in weekly basis.

Number of Days (weekly basis)	Plant Height (cm) (weekly basis)	Plant Leaf Count (weekly basis)	Plant Average Area of Leaves (cm^2^) (weekly basis)	N content (ppm) (weekly basis)	P content (ppm) (weekly basis)	K content (ppm) (weekly basis)
7	0	0	0	44.31	18.12	50.8
7	0	0	0	42.53	18.24	51.05
7	0	0	0	44.81	17.92	50.81
7	0	0	0	42.33	18.57	50.69
….	….	….	….	….	….	….
84	23	14	358	19.15	9.2	27.02
84	23	14	395	19.02	9.4	27.12
84	22	13	387	20.07	8.92	26.18
84	24	12	400	20.45	9.01	26.03

### Primary equation

As the cabbage plant grows, a decrease in major soil nutrients content was observed, concomitant with an increase in plant growth characteristics such as plant height, number of leaves, and average leaf area. These three crucial micronutrients simultaneously influence the enhancement of plant growth characteristics. Consequently, to discern the relationship between these micronutrients and plant growth characteristics, Eq. (1) was formulated [1]. Accordingly, the major soil nutrients of the soil can be predicted based on the plant growth characteristics.

(1) (N+P+K)content=Function(PlantHeight,NumberofLeaves,AverageLeafArea,NumberofDays)

### The DNN model and TanSig function

**Fig. 1:**
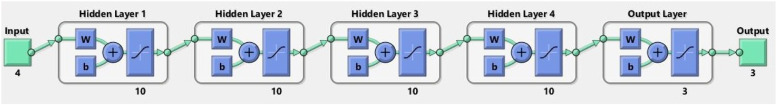
Architecture of the DNN Model.

A total of four hidden layers, each crafted with ten nodes, are included in the meticulously designed neural network architecture tailored specifically for this task, and the tansig transfer function was employed as the activation function. w is the weights and b is the neuron's bias which are represented in Fig. 1. Positioned between the input and output layers, these hidden layers play a crucial role in empowering the network to discern intricate patterns and relationships inherent in the dataset [1,5].

The input layer, strategically configured with four nodes, is dedicated to accommodating key input factors within a weekly basis dataset. These factors include the number of days, the height of the cabbage plant, the number of cabbage leaves, and the average area of cabbage leaves.

**Fig. 2:**
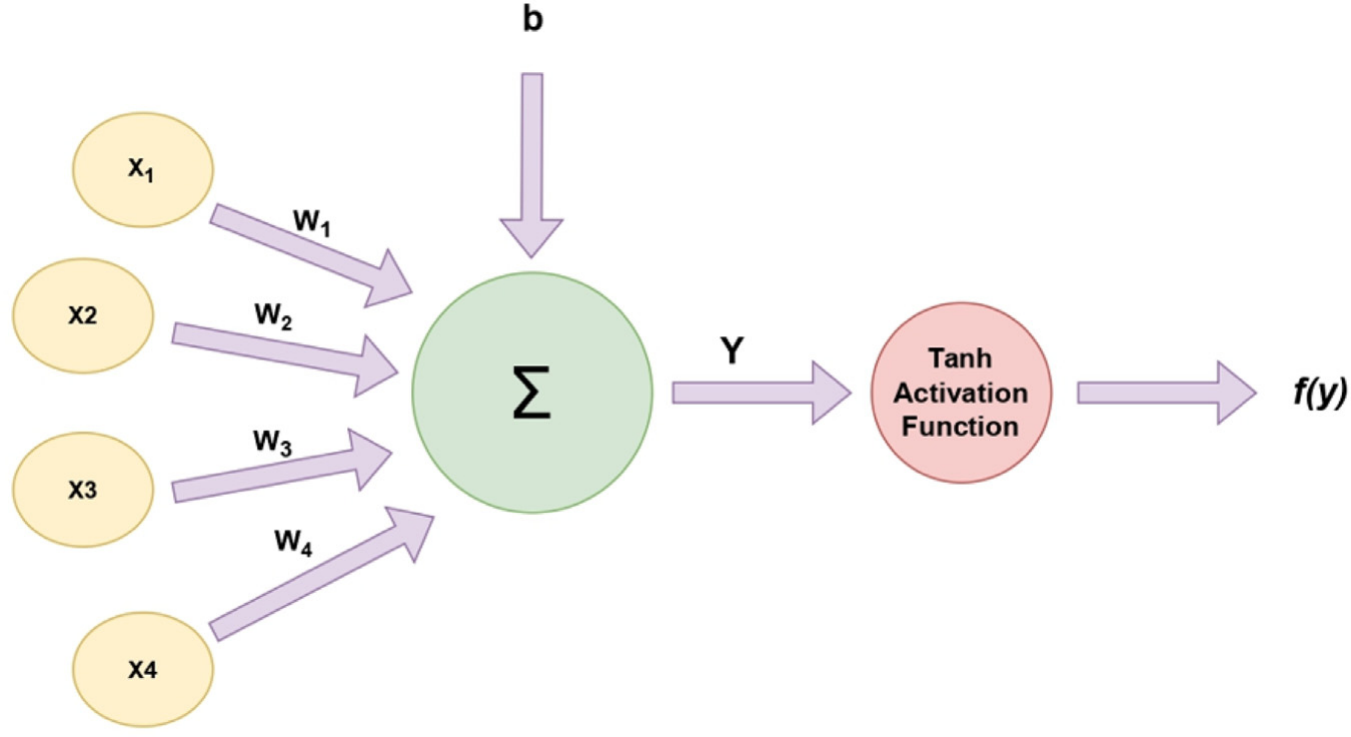
Operational mechanism of the tanh activation function.

The hyperbolic tangent sigmoid transfer function, often denoted as *tansig*, is a non-linear activation function frequently employed in neural networks. However, *tanh* may provide greater accuracy and is recommended for applications that necessitate the use of the hyperbolic tangent function. The *tanh* function is known for its nonlinear nature and produces output values that fall within the range of [−1, 1] [6,7]. Notably, the gradient of the *tanh* function tends to be sharper than that of the sigmoid function [7]. The operational mechanism of tanh activation function is diagrammatically illustrated in Fig. 2, where the input features are denoted as x, output as f(y) the weights as w, and the bias of the neuron as b. In addition to that, the activation function, denoted as f, is employed at the value of each neuron, determining whether the neuron is active or not [8].

The mathematical expression defining the tanh function is given by Eq. (2).

x−Inputmetrics

$$tanh\left(x\right)=\frac{e^{x}-e^{-x}}{e^{x}+e^{-x}}=\frac{1-e^{-2x}}{1+e^{-2x}}=\frac{2-\left(1+e^{-2x}\right)}{1+e^{-2x}}=\frac{2}{1+e^{-2x}}-1$$

(2) tanh(x)=2s(2x)−1

The *tansig* function is calculated as the ratio between the hyperbolic sine and hyperbolic cosine functions. These sigmoidal transfer functions find extensive utilization in the hidden layers of neural networks. Sigmoidal transfer functions, characterized by their S-shaped curve, contribute to the non-linear activation of neurons, enhancing the capacity of ANNs to capture intricate patterns and relationships within data [9,10].

### The connection between the input and output of thetanh function

The tanh activation function aligns with the criteria of being both nonlinear and differentiable [11]. Its differentiability concerning the respective inputs metrics (x) and outputs metrics (y) can be expressed by Eq. (3).

$$\frac{dy}{dx}\;=\frac{d}{dx}tanh\left(x\right)$$

$$\;=\frac{d}{dx}\;\left(\frac{e^{x}-e^{-x}}{e^{x}+e^{-x}}\right)$$

$$\;=\frac{e^{x}+e^{-x}}{{\left(e^{x}+e^{-x}\right)}^{2}}d\left(e^{x}-e^{-x}\right)-\frac{e^{x}-e^{-x}}{{\left(e^{x}+e^{-x}\right)}^{2}}d\left(e^{x}+e^{-x}\right)$$

$$\;=\frac{\left(e^{x}+e^{-x}\right)\left(e^{x}+e^{-x}\right)-\left(e^{x}-e^{-x}\right)\left(e^{x}-e^{-x}\right)}{{\left(e^{x}+e^{-x}\right)}^{2}}$$

$$\;=\frac{{\left(e^{x}+e^{-x}\right)}^{2}-{\left(e^{x}-e^{-x}\right)}^{2}}{{\left(e^{x}+e^{-x}\right)}^{2}}$$

$$\;=\frac{{\left(e^{x}+e^{-x}\right)}^{2}}{{\left(e^{x}+e^{-x}\right)}^{2}}-\frac{{\left(e^{x}-e^{-x}\right)}^{2}}{{\left(e^{x}+e^{-x}\right)}^{2}}$$

$$\;=1-{\left(\frac{e^{x}-e^{-x}}{e^{x}+e^{-x}}\right)}^{2}$$

$$\;=1-{\left(\tanh\left(x\right)\right)}^{2}$$

(3) $$\;=1-{\left(dy\right)}^{2}$$

## Method validation

### Calculation of r and MSE

The following section exhibits an example for the calculation of r and MSE for the developed model. Notations are explained for the calculation as the following.

*S_x_* - Standard deviation of predicted values

*x*_1_ - Value of each predicted values

$\bar{x}$- Average of predicted values

S_y_ - Standard deviation of actual values

*y*_1_ - Value of each actual values

$\bar{y}$ - Average of actual values

*n* - Number of observations

*k* - Number of explanatory variables

$$S_{x}=\sqrt{\frac{\sum {\left(x_{1}-\bar{x}\right)}^{2}}{n-1}}$$

$$\;\sqrt{\frac{,191,61.,464,72}{,130,-1}}$$

= 12.187

$$S_{y}=\sqrt{\frac{\sum {\left(y_{1}-\bar{y}\right)}^{2}}{n-1}}$$

$$\;=\sqrt{\frac{\sum {\left(8.457300275-24.96418733\right)}^{2}+{\left(9.008264463-24.96418733\right)}^{2}+...+{\left(45.26170799-24.96418733\right)}^{2}}{130-1}}$$

$$\;\sqrt{\frac{,177,81.,308,81}{,130,-1}}$$

= 11.740

$$r=\frac{1}{n-1}\;\sum \left(\frac{x_{1}-\bar{x}}{S_{x}}\right).\left(\frac{y_{1}-\bar{y}}{S_{y}}\right)$$

$$\;=\frac{1}{n-1}.\frac{1}{S_{x}.S_{y}}\;\sum \left(x_{1}-\bar{x}\right)\left(y_{1}-\bar{y}\right)$$

$$\;=\frac{1}{130-1}\times \frac{1}{12.187\;\times \;11.740}\;[\left(8.099635256-24.60384444\right)\left(8.457300275-24.96418733\right)+\ldots$$

+(42.16892131−24.60384444)(45.26170799−24.96418733)]

=0.9955

n - Number of items

k - Number of explanatory variables

y - Actual value

$$\hat{y}-\text{Predicted}\;\text{value}$$

Residual = *y* - ŷ

$$MSE=\frac{1}{n}\sum {\left(y-\hat{y}\right)}^{2}$$

$$MSE=\frac{\sum {\left(y-\hat{y}\right)}^{2}}{n-\left(k+1\right)}$$

$$\;\;=\frac{{\left(8.099635256-8.457300275\right)}^{2}+{\left(7.765978731-9.008264463\right)}^{2}\ldots +{\left(42.16892131-45.26170799\right)}^{2}}{130-\left(1+1\right)}$$

$$\;\;=\frac{208.6325792}{128}$$

=1.6065

### Actual vs predicted soil nutrient values under TanSig function

**Fig. 3:**
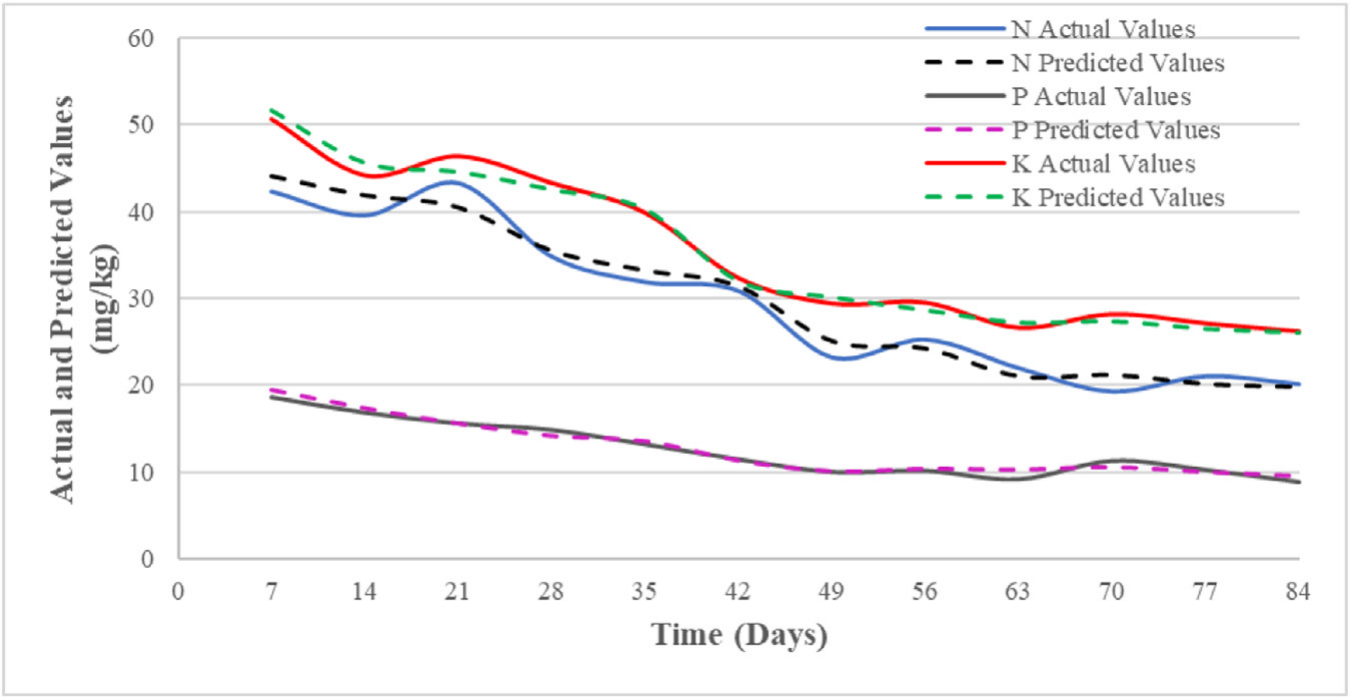
Actual and predicted soil nutrient values using TanSig transfer functions.

In Fig. 3, a detailed comparison is presented between the major soil nutrient values predicted by the DNN model and the actual values. This comparison reflects the accuracy of predictions made by the model using the tansig function. For this graph, a variety of randomly chosen plant growth details were included. The actual and predicted values for P and K are similar on most days, with only small differences observed between the actual and predicted values for N compared to the P and K lines. Overall, the tansig function has provided better prediction results.

## Conclusions

This study has offered a comprehensive justification, both theoretically and mathematically, for the appropriateness of the *tansig* activation function. The sample calculations, followed by a series of additional calculations, have justified the selection of the *tansig* activation function by establishing a solid mathematical foundation for the r and MSE. Furthermore, it was clearly articulated that, in line with this justification, the *tansig* function is deemed the most suitable activation function for the dataset, harmonizing effectively with both actual and predicted soil nutrient values.

## Limitations

The method is demonstrated exclusively for the Green Coronet cabbage variety, and the collected data may vary depending on the climatic factors and soil conditions of the cultivation environment. Therefore, the applicability of this model is limited to a global approach. In addition, the model can be further develop to understand the nutirent requirement for other vegitables with the relevant data.

## Ethics statements

This work does not use the human subjects, Animals and data collected through social media as research materials.

## CRediT authorship contribution statement

**Thilina Abekoon:** Methodology, Writing – original draft. **Hirushan Sajindra:** Software, Formal analysis, Writing – original draft. **B.L.S.K. Buthpitiya:** Data curation. **Namal Rathnayake:** Validation, Writing – review & editing. **D.P.P. Meddage:** Validation, Writing – review & editing. **Upaka Rathnayake:** Conceptualization, Writing – review & editing.

## Declaration of competing interest

The authors declare that they have no known competing financial interests or personal relationships that could have appeared to influence the work reported in this paper.

## Data Availability

Data will be made available on request.
